# Acylphloroglucinol Derivatives from *Garcinia multiflora* with Anti-Inflammatory Effect in LPS-Induced RAW264.7 Macrophages

**DOI:** 10.3390/molecules23102587

**Published:** 2018-10-10

**Authors:** Lin-Yang Cheng, Yun-Chen Tsai, Shu-Ling Fu, Ming-Jen Cheng, Ping-Jyun Sung, Mei-Ing Chung, Jih-Jung Chen

**Affiliations:** 1 School of Pharmacy, College of Pharmacy, Kaohsiung Medical University, Kaohsiung 807, Taiwan; u100830009@kmu.edu.tw; 2 Institute of Traditional Medicine, National Yang-Ming University, Taipei 112, Taiwan; tyc202006@gmail.com (Y.-C.T.); slfu@ym.edu.tw (S.-L.F.); 3 Bioresource Collection and Research Center (BCRC), Food Industry Research and Development Institute (FIRDI), Hsinchu 300, Taiwan; cmj@firdi.org.tw; 4 National Museum of Marine Biology and Aquarium, Pingtung 944, Taiwan; pjsung@nmmba.gov.tw; 5 Faculty of Pharmacy, School of Pharmaceutical Sciences, National Yang-Ming University, Taipei 112, Taiwan; 6 Department of Medical Research, China Medical University Hospital, China Medical University, Taichung 404, Taiwan

**Keywords:** *Garcinia multiflora*, Guttiferae, structure elucidation, nuclear factor κB, nitric oxide, anti-inflammatory activity

## Abstract

Two new acylphloroglucinol derivatives, 13,14-didehydroxygarcicowin C (**1**) and 13,14-didehydroxyisoxanthochymol (**2**), have been isolated from the stems of *Garcinia multiflora*, together with seven known compounds (**3**–**9**). The structures of new compounds **1** and **2** were elucidated by MS and extensive 1D/2D NMR spectroscopic analyses. Among the isolates, 13,14-didehydroxy-isoxanthochymol (**2**) and sampsonione B (**3**) exhibited inhibition against lipopolysaccharide (LPS)-induced NF-κB activation in macrophages at 30 μM with relative luciferase activity values (inhibitory %) of 0.75 ± 0.03 (24 ± 4%) and 0.12 ± 0.03 (88 ± 4%), respectively. Additionally, sampsonione B (**3**) reduced LPS-induced nitric oxide (NO) production in murine RAW264.7 macrophages and did not induce cytotoxicity against RAW 264.7 cells after 24 h treatment. Compound **3** is worth further investigation and may be expectantly developed as an anti-inflammatory *drug candidate.*

## 1. Introduction

*Garcinia multiflora* Champ. (Guttiferae) is a small evergreen tree [1], usually growing 5–15 m tall, distributed in South China, Taiwan, and Hong Kong. Fruit from this plant is edible. In Taiwan, the genus *Garcinia* is represented by three species, *Garcinia multiflora*, *Garcinia linii*, and *Garcinia subelliptica*. Xanthones [2,3,4,5], acylphloroglucinols [6,7,8,9], flavanones [10], and their derivatives are distributed in plants of the genus *Garcinia*. Multiple activities have been reported for these derivatives such as cytotoxic [3,4,5], anti-microbial [2], anti-inflammatory [6,7,8], anti-oxidant [2], and AChE enzymes inhibitory activities [10]. Aberrant inflammation is associated with many diseases such as arthritis, asthma, and cancer [11]. Among immune cells, macrophages are highly responsive to lipopolysaccharide (LPS) and activated macrophages produce multiple pro-inflammatory molecules (such as nitric oxide (NO)). Nuclear factor κB (NF-κB) [12,13] is a transcription factor mediating inflammatory responses and known as a drug target for anti-inflammatory strategy. In our research on the anti-inflammatory constituents of Formosan plants, numerous species had been screened for inhibitory activity on LPS-induced NF-κB activation, and *G. multiflora* was found to be an active species. Phytochemical investigations on the stems of *G. multiflora* has resulted in the isolation of two new acylphloroglucinol derivatives, 13,14-didehydroxygarcicowin C (**1**) and 13,14-didehydroxy-isoxanthochymol (**2**), along with seven known compounds. We evaluated the anti-inflammatory effect of the isolated compounds in LPS-stimulated RAW264.7 macrophages and found that 13,14-didehydroxyisoxanthochymol (**2**) and sampsonione B (**3**) decreased NF-κB activity. Moreover, sampsonione B (**3**) inhibited the production of nitric oxide (NO) in LPS-activated macrophages. In this article, the structural elucidation of **1** and **2** and the inhibitory activity of the isolates on LPS-induced NF-κB activation are described.

## 2. Results and Discussion

### 2.1. Isolation and Structural Elucidation

**Figure 1:**
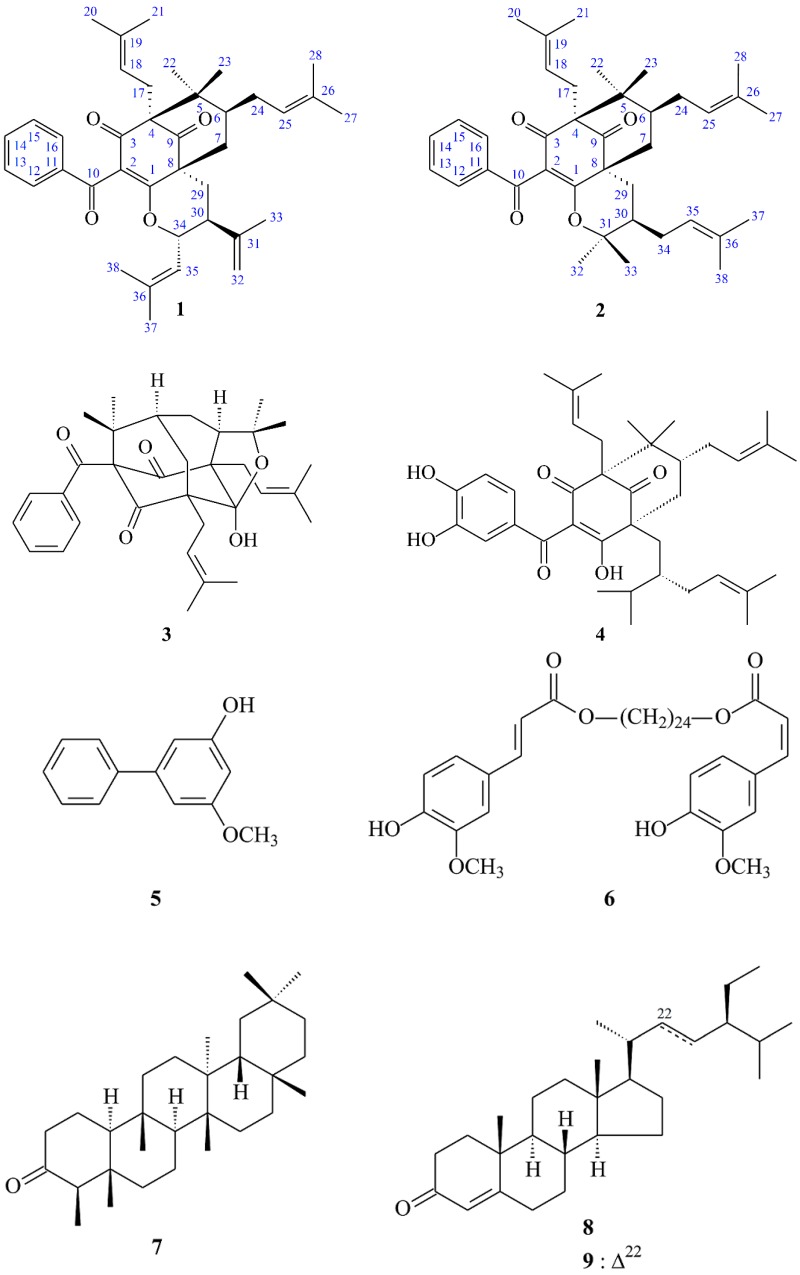
The chemical structures of compounds **1**–**9** isolated from *Garcinia multiflora*.

Chromatographic isolation and purification of the EtOAc-soluble fraction of a MeOH extract of stems of *G. multiflora* on a silica gel column and preparative thin-layer chromatography (TLC) obtained two new (**1** and **2**) and seven known compounds (**3**–**9**) (Figure 1).

**Figure 2:**
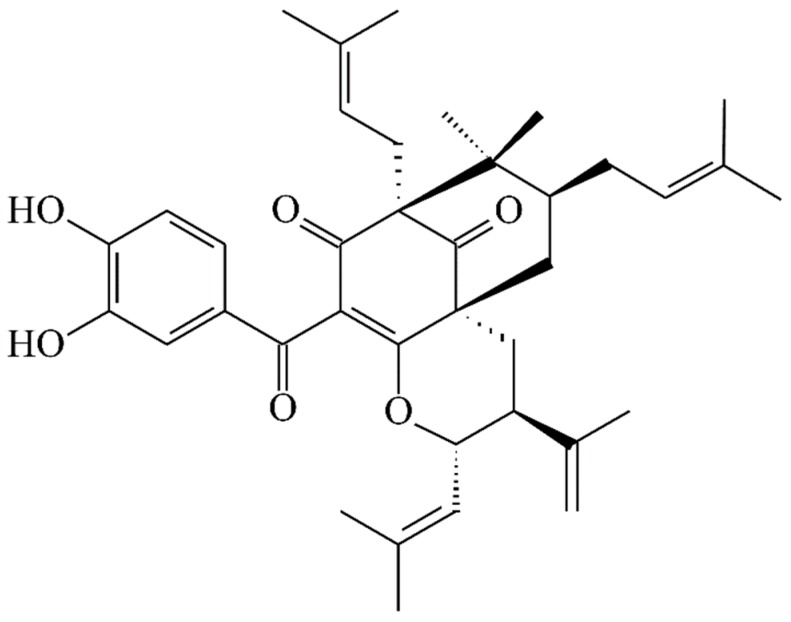
The chemical structure of garcicowin C.

**Figure 3:**
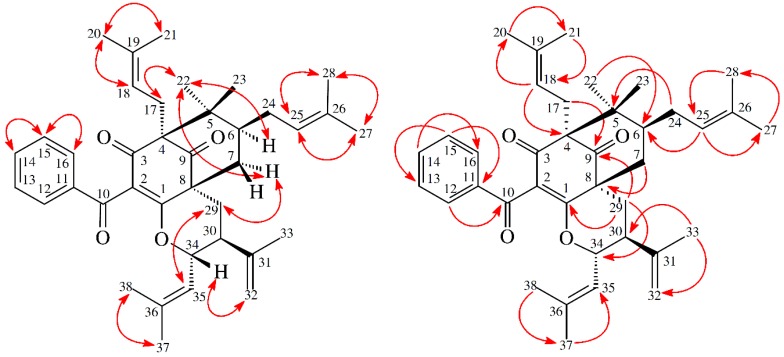
Key NOESY (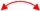) and HMBC (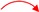) correlations of **1**.

**Figure 4:**
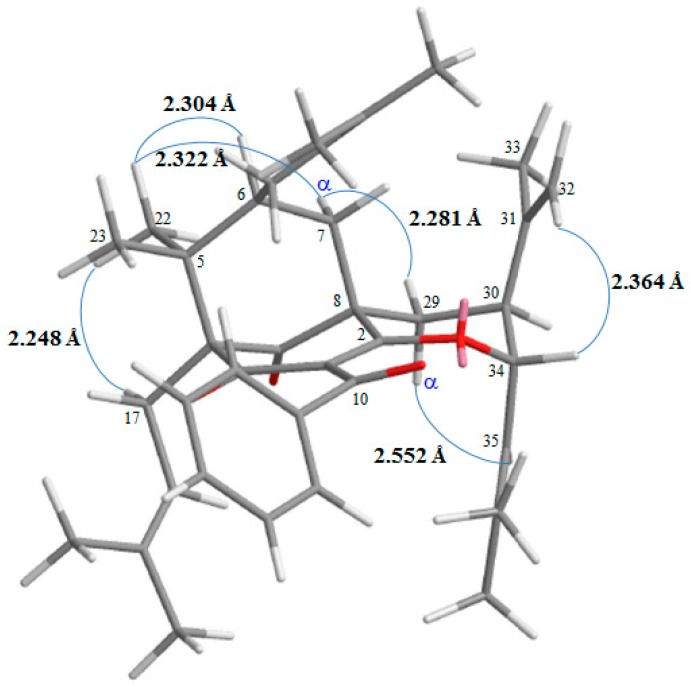
Selected NOESY correlations and relative configuration of **1**.

13,14-Didehydroxygarcicowin C (**1**) was obtained as colorless, amorphous powder. The ESI-MS (Figure S1) displayed the quasi-molecular ion [M + H]^+^ at *m*/*z* 569, implying a molecular formula of C_38_H_49_O_4_, which was confirmed by the HR-ESI-MS (*m*/*z* 569.36284 [M + H]^+^, calcd 569.36254) (Figure S2) and by the ^1^H, ^13^C, and DEPT NMR data. The presence of carbonyl groups was revealed by the bands at 1729, 1682, and 1645 cm^−1^ in the IR spectrum and was confirmed by signals at δ_C_ 209.0, 193.8, and 193.1 in the ^13^C NMR spectrum. The ^1^H and ^13^C NMR spectrum (Table 1) (Figures S3 and S4) of **1** showed signals for an acylphloroglucinol derivative based on the presence of a 2,2-dimethylbicyclo[3.3.1]nonane ring system, a benzoyl group, two isoprenyl groups, and another C_10_ unit (C-29 through C-38). The ^1^H NMR data of **1** were similar to those of garcicowin C (Figure 2) [14], except that the benzoyl group [δ_H_ 7.38 (2H, t, *J* = 7.5 Hz, H-13, and H-15), 7.50 (1H, t, *J* = 7.5 Hz, H-14), and 7.77 (2H, d, *J* = 7.5 Hz, H-12, and H-16)] of **1** replaced the 3,4-dihydroxybenzoyl group of garcicowin C. This was supported by the HMBC correlations observed between H-12 (δ_H_ 7.77) and C-10 (δ_C_ 193.1), C-14 (δ_C_ 133.0), and C-16 (δ_C_ 128.7). The relative configuration of **1** was deduced from the NOESY cross-peaks (Figure 3) of H-17/H-22, H-22/H-6, H_α_-7/H-22, H_α_-7/H-29, H_α_-29/H-35, and H-34/H-32. Consequently, H-6, the isoprenyl group at C-4, and the bond between C-8 and C-29 are on the α-side, and H-34 and the prop-1-en-2-yl group at C-30 are on the β-side of **1**. According to the data of the ^1^H–^1^H COSY (Figure S5) and NOESY (Figure S6) spectra, a computer-created 3D structure was established by applying the above-mentioned molecular modeling program with MM2 force-field calculations for energy minimization. The NOESY experiment of **1** showed selected cross-peaks as shown in the 3D drawing (Figure 4). The calculated distances between H-17/H-22 (2.248 Å), H-22/H-6 (2.304 Å), H_α_-7/H-29 (2.281 Å), H_α_-29/H-35 (2.552 Å), and H-34/H-32 (2.364 Å) are all less than 4.00 Å; this corresponds with the well-defined NOESY examined for each of the proton pairs. The absolute configuration of **1** was indicated by CD Cotton effects at 311 (Δε + 2.1), 267 (Δε − 8.5), 223 (Δε + 5.2) nm in analogy with garcicowin C [15]. The full assignment of ^13^C and ^1^H NMR resonances was substantiated by DEPT, ^1^H–^1^H COSY, NOESY (Figure 3), HMBC (Figure 3) (Figure S7), and HSQC (Figure S8) experiments. On the basis of the above evidence, the structure of **1** was established as 13,14-didehydroxygarcicowin C.

**Figure 5:**
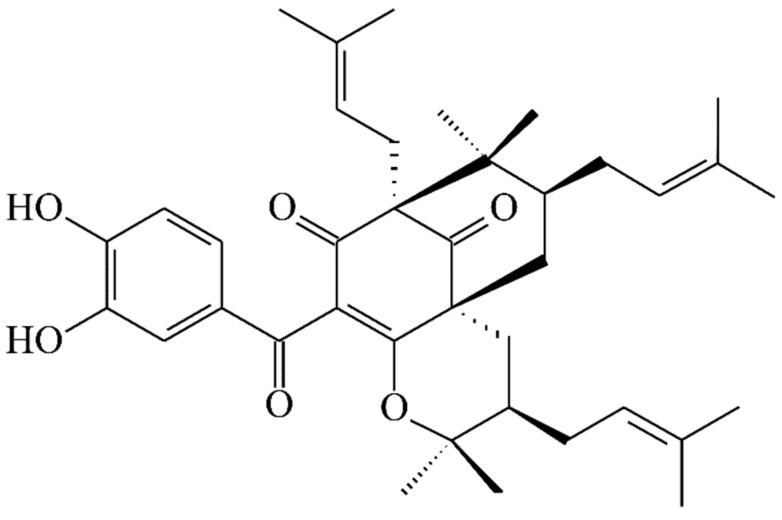
The chemical structure of isoxanthochymol.

**Figure 6:**
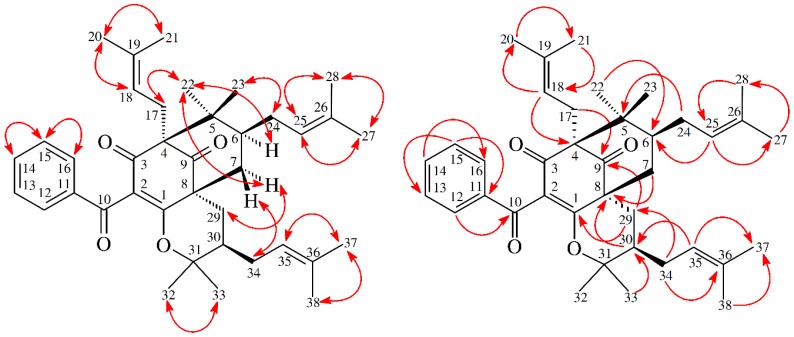
Key NOESY (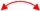) and HMBC (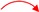) correlations of **2**.

**Figure 7:**
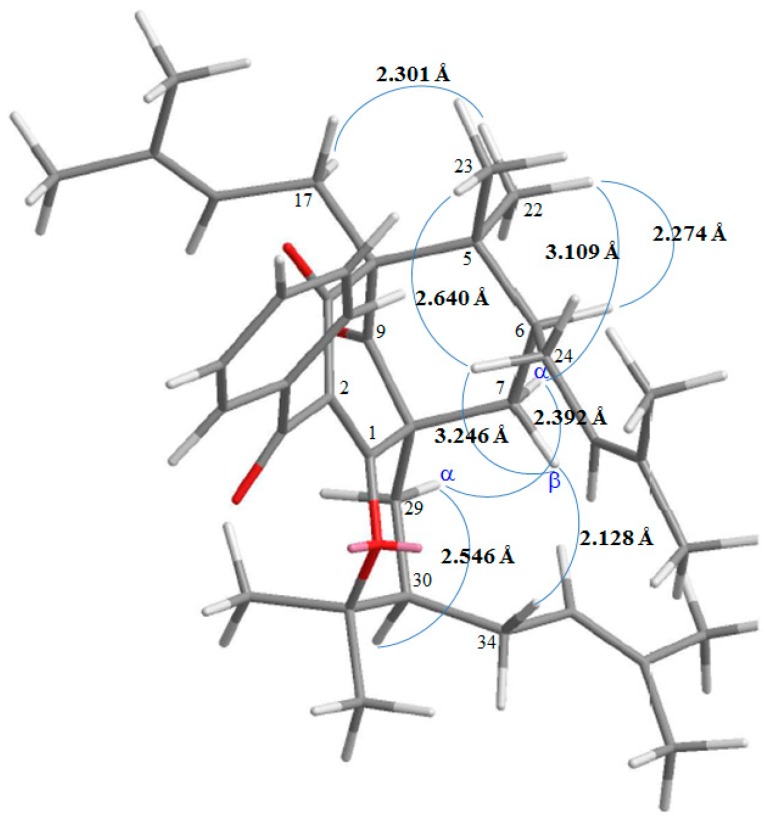
Selected NOESY correlations and relative configuration of **2**.

13,14-Didehydroxyisoxanthochymol (**2**) was isolated as a colorless amorphous powder with molecular formula C_38_H_50_O_4_ as established by ESI-MS (Figure S9) and HR-ESI-MS (Figure S10), revealing an [M + H]^+^ ion at *m*/*z* 571.37822 (calcd for C_38_H_51_O_4_, 571.37819). The presence of carbonyl groups was revealed by the bands at 1724, 1675, and 1637 cm^−1^ in the IR spectrum and was confirmed by signals at δ_C_ 207.1, 193.8, and 193.7 in the ^13^C NMR spectrum. The ^1^H- and ^13^C NMR spectrum (Table 1) (Figures S11 and S12) of **2** showed signals for an acylphloroglucinol derivative based on the presence of a 2,2-dimethylbicyclo[3.3.1]nonane ring system, a benzoyl group, three isoprenyl groups, and another C_5_ unit (C-29 through C-33). The NMR data of **2** was similar to those of isoxanthochymol (Figure 5) [16], except that the benzoyl group [δ_H_ 7.36 (2H, t, *J* = 7.5 Hz, H-13 and H-15), 7.49 (1H, t, *J* = 7.5 Hz, H-14), and 7.72 (2H, d, *J* = 7.5 Hz, H-12 and H-16)] of **2** replaced the 3,4-dihydroxybenzoyl group of isoxanthochymol [16]. This was supported by the HMBC correlations observed between H-12 (δ_H_ 7.72) and C-10 (δ_C_ 193.8), C-14 (δ_C_ 132.9), and C-16 (δ_C_ 128.8). The relative configuration of **2** was determined by NOESY cross-peaks (Figure 6) of H-17/H-22, H-22/H-6, H_α_-7/H-22, H_α_-7/H-29, H_α_-29/H-30, and H_β_-7/H-34. Consequently, H-6, the isoprenyl group at C-4, and the bond between C-8 and C-29 are on the α-side, and the isoprenyl group at C-6 and the isoprenyl group at C-30 are on the β-side of **2**. A computer-created 3D structure (Figure 7) was established by applying the above-mentioned molecular modeling program with MM2 force-field calculations for energy minimization. The calculated distances between H-17/H-22 (2.301 Å), H-22/H-6 (2.274 Å), H-22/H_α_-7 (3.109 Å), H_α_-29/H-30 (2.546 Å), H_β_-7/H-34 (2.128 Å), and H_β_-7/H-24 (3.246 Å) are all less than 4.00 Å; this corresponds with the well-defined NOESY observed for each of the proton pairs. The absolute configuration of **2** was confirmed by the similar CD Cotton effects [270 (Δε + 13.6), 224 (Δε − 8.4) nm] compared with analogous benzoylphloroglucinol derivative, isoxanthochymol [16]. The structure elucidation of **2** was confirmed by ^1^H–^1^H COSY (Figure S13) and NOESY (Figure 6) (Figure S14) techniques and ^13^C NMR assignments were supported by DEPT, HMBC (Figure 6) (Figure S15), and HSQC (Figure S16) experiments. According to the above evidence, the structure of **2** was established as 13,14-didehydroxyisoxanthochymol.

### 2.2. Structure Identification of the Known Isolates

The known isolated compounds were easily identified by a comparison of spectroscopic and physical data (^1^H NMR, UV, MS, [α]_D_, and IR) with corresponding authentic samples or literature values, and this included two acylphloroglucinol derivatives, sampsonione B (**3**) [17] and garcinol (**4**) [16], a biphenyl derivative, 3-hydroxy-5-methoxybiphenyl (**5**) [18], a ferulic acid ester derivative, 1,24-tetracosanediol diferulate (**6**) [19], a triterpene, friedelan-3-one (**7**) [20], and a mixture of steroids, β-sitostenone (**8**) [21] and stigmasta-4,22-dien-3-one (**9**) [22].

### 2.3. Biological Studies

**Figure 8:**
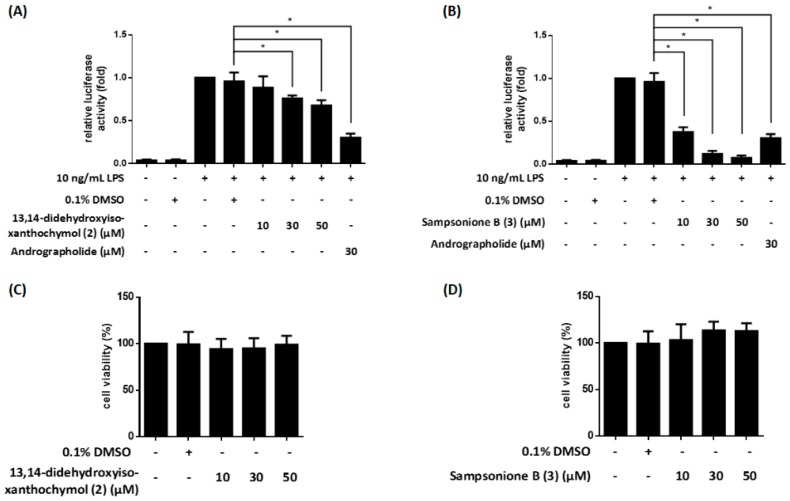
The compounds **2** and **3** inhibit NF-κB activation in lipopolysaccharide (LPS)-induced RAW 264.7/Luc-P1 cells. The luciferase activities of 13,14-didehydroxyisoxanthochymol (**2**) (**A**) and sampsonione B (**3**) (**B**) in LPS-stimulated RAW 264.7/Luc-P1 macrophages were observed. Andrographolide is the positive control. The cell viability of RAW 264.7/Luc-P1 cells incubated with 13,14-didehydroxyisoxanthochymol (**2**) (**C**) or sampsonione B (**3**) (**D**) for 24 h was measured using MTT assay. * indicates significant difference vs. LPS-treated vehicle control (*p* < 0.05).

NF-κB [12,13] plays an essential role in inflammatory responses. We previously established an LPS-responsive macrophage cell clone (RAW264.7/Luc-P1), in which NF-κB activity correlates with the luciferase gene expression [23]. The RAW 264.7/Luc-P1 cells allowed us to successfully identify NF-κB-suppressing compounds such as fisetin and methyl isornate [24,25]. Therefore, we applied this system to measure the effects of isolated compounds on NF-κB activity, and their inhibitory activities (with inhibitory percentages) are summarized in Table 2. 13,14-Didehydroxyisoxantho-chymol (**2**) and sampsonione B (**3**) show significant inhibition of LPS-stimulated NF-κB activity (Figure 8A,B). Moreover, 13,14-didehydroxyisoxanthochymol (**2**) and sampsonione B (**3**) did not induce cytotoxicity against RAW 264.7/Luc-P1 cells after 24 h treatment (Figure 8C,D).

**Figure 9:**
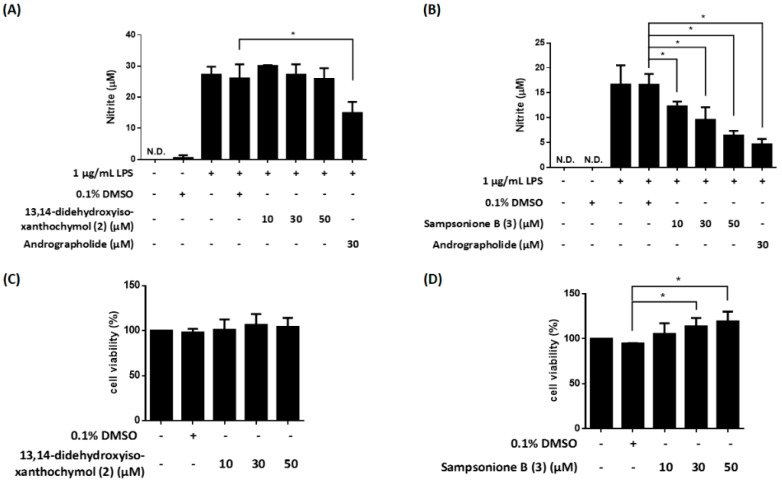
The effects of compounds **2** and **3** on nitric oxide (NO) production in LPS-induced RAW 264.7 macrophages. The effects of 13,14-didehydroxyisoxanthochymol (**2**) (**A**) and sampsonione B (**3**) (**B**) in LPS-treated RAW 264.7 macrophages were detected by Griess reagent. Andrographolide is the positive control. The cell viability of RAW 264.7 cells incubated with 13,14-didehydroxy- isoxanthochymol (**2**) (**C**) or sampsonione B (**3**) (**D**) for 24 h was measured using MTT assay. * indicates significant difference vs. LPS-treated vehicle control (*p* < 0.05).

LPS-mediated NF-κB activation results in upregulation of pro-inflammatory molecules, such as NO, in macrophages [12,26]. Thus, NO generation is a hallmark of inflammatory responses. Our study further evaluated the potential anti-inflammatory compounds, 13,14-didehydroxy- isoxanthochymol (**2**) and sampsonione B (**3**) on NO production. The result showed that 13,14-didehydroxyisoxanthochymol (**2**) did not obviously affect LPS-induced NO generation in RAW264.7 macrophages and did not display cytotoxicity against RAW 264.7 cells after 24 h treatment (Figure 9A,C). In contrast, sampsonione B (**3**) could suppress LPS-induced NO generation in a concentration-dependent manner (Figure 9B) without causing significant cytotoxicity (Figure 9D). 

It is observed that the inhibition on NO production is in a close correlation with NF-κB activation (e.g. compound **3** in Figure 8B and Figure 9B). Thus, compound **3** may be involved in NF-κB-dependent NO regulation.

## 3. Experimental Section

### 3.1. General Procedures

Ultraviolet (UV) spectra were measured on a Jasco UV-240 spectrophotometer (Jasco Co., Hachioji, Japan). Optical rotations were measured using a Jasco DIP-370 polarimeter (Jasco Co., Hachioji, Japan) in MeOH. CD spectra were obtained on a Jasco J-815 spectropolarimeter (Jasco Co., Hachioji, Japan). Infrared (IR) spectra (neat or KBr) were determined on a Perkin Elmer 2000 FT-IR spectrometer (Perkin-Elmer Corp., Waltham, MA, USA). Nuclear magnetic resonance (NMR) spectra, including nuclear Overhauser effect spectrometry (NOESY), correlation spectroscopy (COSY), heteronuclear single-quantum coherence (HSQC), and heteronuclear multiple-bond correlation (HMBC) experiments, were measured on a Varian Inova 500 spectrometer (Varian, Palo Alto, CA, USA) operating at 125 MHz (^13^C) and 500 MHz (^1^H), respectively, with chemical shifts given in ppm (δ) using tetramethylsilane (TMS) as an internal standard. Electrospray ionization (ESI) and high-resolution electrospray ionization (HRESI)-mass spectra were determined on a Bruker APEX II mass spectrometer (Bruker, Billerica, MA, USA). Silica gel 60 F-254 (Merck, Darmstadt, Germany) was used for preparative thin-layer chromatography (PTLC) and thin-layer chromatography (TLC). Silica gel (70–230 and 230–400 mesh, Merck) was used for column chromatography (CC).

### 3.2. Plant Material

The stems of *G. multiflora* was collected from Mudan, Pingtung County, Taiwan, in December 2012 and identified by Dr. M. H. Yen (School of Pharmacy, College of Pharmacy, Kaohsiung Medical University, Taiwan). A voucher specimen (GM-201212) was deposited in the Faculty of Pharmacy, School of Pharmaceutical Sciences, National Yang-Ming University, Taipei, Taiwan.

### 3.3. Extraction and Isolation

The dried stems of *G. multiflora* (5.4 kg) were extracted three times with MeOH (10 L each) for 3 days. The MeOH extracts were concentrated under reduced pressure at 35 °C, and the residue (211 g) was partitioned between EtOAc and H_2_O (1:1). The EtOAc layer was concentrated to give a residue (fraction A, 118 g). The water layer was further extracted with *n*-BuOH, and the water-soluble part (fraction C, 41 g) and the *n*-BuOH-soluble part (fraction B, 45 g) were separated. Fraction A (118 g) was separated on silica gel (70–230 mesh, 5.2 kg), eluting with CH_2_Cl_2_, gradually increasing the polarity with MeOH to give 13 fractions: A1 (2 L, CH_2_Cl_2_), A2 (2 L, CH_2_Cl_2_/MeOH, 90:1), A3 (2 L, CH_2_Cl_2_/MeOH, 80:1), A4 (1 L, CH_2_Cl_2_/MeOH, 50:1), A5 (1 L, CH_2_Cl_2_/MeOH, 40:1), A6 (2 L, CH_2_Cl_2_/MeOH, 30:1), A7 (2 L, CH_2_Cl_2_/MeOH, 20:1), A8 (5 L, CH_2_Cl_2_/MeOH, 10:1), A9 (7 L, CH_2_Cl_2_/MeOH, 5:1), A10 (2 L, CH_2_Cl_2_/MeOH, 4:1), A11 (4 L, CH_2_Cl_2_/MeOH, 2:1), A12 (5 L, CH_2_Cl_2_/MeOH, 1:1), and A13 (5 L, MeOH).

Fraction A2 (5.6 g) was chromatographed further on silica gel (70–230 mesh, 250 g) eluting with *n*-hexane/acetone (20:1–0:1) to give 10 fractions (each 1.2 L, A2-1−A2-10). Compound **7** (7.2 mg) was yielded from fraction A2-1 (85 mg) by recrystallization with *n*-hexane/EtOAc. Compounds **8** and **9** (12.2 mg) were obtained from fraction A2-2 (358 mg) by recrystallization with *n*-hexane/EtOAc. Fraction A2-4 (126 mg) was purified further by preparative TLC (silica gel, CH_2_Cl_2_) to obtain **1** (5.2 mg) and **2** (2.3 mg). Part (168 mg) of fraction A2-5 was purified by preparative TLC (silica gel, *n*-hexane/EtOAc, 6:1) to obtain **3** (2.2 mg). Part (120 mg) of fraction A2-7 was purified by preparative TLC (silica gel, *n*-hexane/EtOAc, 3:1) to afford **5** (2.2 mg). Part (89 mg) of fraction A2-9 was purified by preparative TLC (silica gel, CHCl_3_) to obtain **6** (2.5 mg). Fraction A3 (4.5 g) was chromatographed further on silica gel (230–400 mesh, 205 g) eluting with *n*-hexane/EtOAc (15:1–0:1) to give 7 fractions (each 1 L, A3-1–A3-7). Part (172 mg) of fraction A3-5 was purified by preparative TLC (silica gel, *n*-hexane/acetone, 2:1) to afford **4** (6.2 mg).

13,14-didehydroxygarcicowin C (**1**): amorphous powder; [α] ${}_{D}^{25}$ = −68.6 (*c* 0.18, CHCl_3_); CD (MeOH, Δε): 311 (+2.1), 267 (−8.5), 223 (+5.2) nm; UV (MeOH): λ_max_ (log ε) = 250 (3.90), 273 (sh, 3.77) nm; IR (KBr): υ_max_ = 1729 (C=O), 1682 (C=O), 1645 (C=O) cm^−1^; ESI-MS: *m*/*z* = 569 [M + H]^+^; HR-ESI-MS: *m*/*z* = 569.36284 [M + H]^+^ (calcd for C_38_H_49_O_4_, 569.36254); ^1^H and ^13^C NMR data: see Table 1. 

13,14-didehydroxyisoxanthochymol (**2**): amorphous powder; [α] ${}_{D}^{25}$ = +205.7 (*c* 0.15, CHCl_3_); CD (MeOH, Δε): 270 (+13.6), 224 (−8.4) nm; UV (MeOH): λ_max_ (log ε) = 203 (4.16), 249 (3.96), 276 (sh, 4.02) nm; IR (KBr): υ_max_ = 1724 (C=O), 1675 (C=O), 1637 (C=O) cm^−1^; ESI-MS: *m*/*z* = 571 [M + H]^+^; HR-ESI-MS: *m*/*z* = 571.37822 [M + H]^+^ (calcd for C_38_H_51_O_4_, 571.37819); ^1^H and ^13^C NMR data: see Table 1. 

### 3.4. Biological Assay

The effect of the isolates on LPS-induced NF-κB activation in RAW 264.7/Luc-P1 macrophage was assessed by determining the luminescence resulted from luciferase activity in a concentration-dependent manner. The purity of the tested compounds was >98% as identified by MS and NMR.

#### 3.4.1. Cells and Culture Medium

The RAW 264.7/Luc-P1 cell is an LPS-responsive cell line with an integrated reporter gene (pELAM1-Luc) [23]. The murine RAW 264.7 macrophage and RAW 264.7/Luc-P1 cells were cultured and originated conditions as described previously [23,24].

#### 3.4.2. Luciferase Reporter Assay

The RAW 264.7/Luc-P1 cells (1.5 × 10^5^ cells in 24-well plates) were treated with pure compounds, the positive control (30 μM andrographolide) or vehicle (0.1% DMSO) for 1 h and then LPS (10 ng/mL) for 23 h. The treated cells were then collected and assessed using luciferase assays (Promega, Madison, WI, USA) as described previously [25].

#### 3.4.3. 3-(4,5-Dimethylthiazol-2-yl)-2,5-diphenyltetrazolium Bromide (MTT) Assay

RAW 264.7/Luc-P1 cells or RAW 264.7 cells (10^4^ cells in 96-well plates) were treated with 13,14-didehydroxyisoxanthochymol (**2**), sampsonione B (**3**) and 0.1% DMSO for 24 h. MTT assays were performed as described previously [25].

#### 3.4.4. Nitric Oxide (NO) Production

The RAW 264.7 cells (4 × 10^4^ cells in 96-well plates) were treated with 13,14-didehydroxy- isoxanthochymol (**2**), sampsonione B (**3**) and 0.1% DMSO for 1 h and then incubated with LPS (1μg/mL) for 23 h. The 100 μL of cell culture medium with an equal volume of Griess reagent (0.1% naphthylethylenediamine dihydrochloride and 1% sulfanilamide in 2.5% phosphoric acid) in a 96-well plate was incubated for 10 min. The absorbance at 550 nm was measured by using a Model 680 Microplate Reader (Bio-rad, Hercules, CA, USA). The level of NO production was calculated from sodium nitrite (NaNO_2_) standard curve [27].

#### 3.4.5. Statistical Analysis

The data are displayed as mean ± SD from three independent experiments. Statistical analysis was performed using Student’s t test. Differences were considered as statistically significant when *p* < 0.05.

## 4. Conclusions

Our research on the phytochemical investigation of *G. multiflora* has led to the isolation of two new (**1**, **2**) and seven known (**3**–**9**) compounds. The structures of these isolates were established by spectroscopic data. Based on the results of our bioactivity assays, among the isolates, 13,14-didehydroxyisoxanthochymol (**2**) and sampsonione B (**3**) exhibited inhibition against lipopolysaccharide (LPS)-induced NF-κB activation in macrophages at 30 μM with relative luciferase activity values of 0.75 ± 0.03 and 0.12 ± 0.03, respectively. Furthermore, samposonione B (**3**) showed LPS-induced NO generation in concentration dependent manner. Thus, our research suggests *G. multiflora* and its isolated compound (especially **3**) are worth further study and may be expectantly developed as the candidates for the prevention or treatment of diverse inflammatory diseases.

## Figures and Tables

**Table 1:** ^1^H NMR (500 MHz) and ^13^C NMR (125 MHz) data for compounds **1** and **2** in CDCl_3_.

Position	1		2	
	δ_C_, type	δ_H_ (*J* in Hz)	δ_C_, type	δ_H_ (*J* in Hz)
1	170.8, C		171.5, C	
2	129.1, C		125.3, C	
3	193.8, C		193.7, C	
4	69.2, C		68.3, C	
5	46.7, C		46.3, C	
6	46.4, CH	1.51, m	46.2, CH	1.45, m
7	38.1, CH_2_	2.60, d (14.5)	39.5, CH_2_	2.28, d (14.5)
		1.89, dd (14.5, 7.5)		1.98, dd (14.5, 7.0)
8	48.1, C		51.2, C	
9	209.0, C		207.1, C	
10	193.1, C		193.8, C	
11	137.5, C		137.5, C	
12	128.7, CH	7.77, d (7.5)	128.8, CH	7.72, d (7.5)
13	128.3, CH	7.38, t (7.5)	128.3, CH	7.36, t (7.5)
14	133.0, CH	7.50, t (7.5)	132.9, CH	7.49, t (7.5)
15	128.3, CH	7.38, t (7.5)	128.3, CH	7.36, t (7.5)
16	128.7, CH	7.77, d (7.5)	128.8, CH	7.72, d (7.5)
17	25.3, CH_2_	2.66, dd (13.5, 8.0)	25.5, CH_2_	2.68, dd (14.0, 8.5)
		2.47, m		2.43, dd (14.0, 5.0)
18	119.8, CH	4.91, br t (8.0)	119.8, CH	4.95, dd (8.5, 5.0)
19	134.6, C		134.7, C	
20	26.1, CH_3_	1.62, s	26.2, CH_3_	1.62, s
21	18.1, CH_3_	1.56, s	18.0, CH_3_	1.58, s
22	26.8, CH_3_	1.00, s	26.8, CH_3_	0.98, s
23	22.3, CH_3_	1.18, s	22.5, CH_3_	1.17, s
24	29.3, CH_2_	2.53, m	29.3, CH_2_	2.65, m
		2.23, m		2.19, m
25	124.9, CH	4.94, br t (7.5)	124.9, CH	4.90, br t (7.0)
26	133.1, C		133.1, C	
27	26.0, CH_3_	1.71, s	26.0, CH_3_	1.70, s
28	18.2, CH_3_	1.67, s	18.1, CH_3_	1.69, s
29	33.2, CH_2_	2.32, t (14.0)	28.4, CH_2_	3.05, dd (14.0, 3.5)
		1.75, dd (14.0, 2.5)		0.92, m
30	42.8, CH	2.46, m	42.8, CH	1.39, m
31	143.5, C		86.4, C	
32	113.9, CH_2_	4.80, s	28.5, CH_3_	0.83, s
		4.84, s		
33	20.3, CH_3_	1.66, s	21.3, CH_3_	1.23, s
34	79.7, CH	4.20, t (9.0)	29.6, CH_2_	2.03, m
				1.78, m
35	121.4, CH	5.01, br d (9.0)	121.4, CH	5.19, br t (6.5)
36	141.7, C		133.7, C	
37	25.7, CH_3_	1.61 s	25.8, CH_3_	1.77 s
38	17.8, CH_3_	1.10 s	18.1, CH_3_	1.60 s

**Table 2:** The effects of compounds **1**–**7** from the stems of *Garcinia multiflora* on NF-κB activation in RAW 264.7/Luc-P1 cells.

Compounds ^a^	Relative Luciferase Activity	Inhibition (%) ^e^
	Mean ± SD ^d^	Mean ± SD ^d^
13,14-Didehydroxygarcicowin C (**1**)	0.78 ± 0.11	21 ± 11
13,14-Didehydroxyisoxanthochymol (**2**)	0.75 ± 0.03 *	24 ± 4 *
Sampsonione B (**3**)	0.12 ± 0.03 *	88 ± 4 *
Garcinol (**4**)	1.23 ± 0.21	
3-Hydroxy-5-methoxybiphenyl (**5**)	0.85 ± 0.06	14 ± 3
1,24-Tetracosanediol diferulate (**6**)	0.96 ± 0.08	3 ± 10
Friedelan-3-one (**7**)	1.04 ± 0.20	
LPS-treated vehicle control ^b^	0.94 ± 0.09	5 ± 9
Andrographolide ^c^	0.31± 0.05 *	70 ± 5 *

^a^ Compounds **1**–**7**: 30 μM. ^b^ Vehicle control: 0.1% DMSO. ^c^ Andrographolide (30 μM) is the positive control. ^d^ Data are displayed as the mean ± SD from three independent experiments. * indicates significant difference versus lipopolysaccharide (LPS) (1 μg/mL)-treated vehicle control (*p* < 0.05). ^e^ Inhibition (%) = [1 − luciferase activity (compounds)/luciferase activity (LPS-treated control)] × 100.

## Supplementary Materials

Supplementary materials are available online, Figures S1–S8: MS, 1D, and 2D-NMR spectra for 13,14-didehydroxygarcicowin C (**1**), Figures S9–S16: MS, 1D, and 2D-NMR spectra for 13,14-didehydroxyisoxanthochymol (**2**).
