# Supplementary material for: Acylphloroglucinol Derivatives from Garcinia multiflora with Anti-Inflammatory Effect in LPS-Induced RAW264.7 Macrophages
Source: Molecules. 2018 Oct 10;23(10):2587. doi: 10.3390/molecules23102587 (PMC6222856; doi:10.3390/molecules23102587)
Supplement: Supplementary file 1 [file molecules-23-02587-s001.docx]

**Supplementary Materials**

Acylphloroglucinol Derivatives from *Garcinia multiflora* with Anti-inflammatory Effect in LPS-induced RAW264.7 Macrophages

Lin-Yang Cheng ^1^, Yun-Chen Tsai ^2^, Shu-Ling Fu ^2,†^, Ming-Jen Cheng ^3^, Ping-Jyun Sung ^4^,
[Mei-Ing Chung](http://www.mdpi.com/search?authors=Mei-Ing%20Chung&orcid=) ^1,†,^* and [Jih-Jung Chen](http://www.mdpi.com/search?authors=Jih-Jung%20Chen&orcid=) ^5,6,^*

^1^ School of Pharmacy, College of Pharmacy, Kaohsiung Medical University, Kaohsiung 807, Taiwan;
u100830009@kmu.edu.tw (L.-Y.C.)

^2^ Institute of Traditional Medicine, National Yang-Ming University, Taipei 112, Taiwan;
tyc202006@gmail.com (Y.-C.T.); [slfu@ym.edu.tw](mailto:slfu@ym.edu.tw) (S.-L.F.)

^3^ Bioresource Collection and Research Center (BCRC), Food Industry Research and Development Institute (FIRDI), Hsinchu 300, Taiwan; cmj@firdi.org.tw

^4^ National Museum of Marine Biology and Aquarium, Pingtung 944, Taiwan; [pjsung@nmmba.gov.tw](mailto:pjsung@nmmba.gov.tw)

^5^ Faculty of Pharmacy, School of Pharmaceutical Sciences, National Yang-Ming University, Taipei 112, Taiwan

^6^ Department of Medical Research, China Medical University Hospital, China Medical University, Taichung 404, Taiwan

***** Correspondence: [chenjj@ym.edu.tw](mailto:chenjj@ym.edu.tw) (J.-J.C.); Tel.: +886-2-2826-7195; [meinch@kmu.edu.tw](mailto:meinch@kmu.edu.tw) (M.-I.C.);
Tel.: +886-7-312-1101 (ext. 2672)

† Authors have contributed equally in this manuscript.

Contents

**Figure S1.** ESI-MS spectrum of **1**.................................................................................................... S3

**Figure S2.** HR-ESI-MS spectrum of **1**............................................................................................. S3

**Figure S3.** ^1^H-NMR spectrum (CDCl_3_, 500 MHz) of **1**................................................................... S4

**Figure S4.** ^13^C-NMR spectrum (CDCl_3_, 125 MHz) of **1**.................................................................. S4

**Figure S5.** ^1^H-^1^H COSY spectrum of **1**.............................................................................................S5

**Figure S6.** NOESY spectrum of **1**.................................................................................................... S5

**Figure S7.** HMBC spectrum of **1**..................................................................................................... S6

**Figure S8.** HSQC spectrum of **1**..................................................................................................... S6

**Figure S9.** ESI-MS spectrum of **2**.................................................................................................... S7

**Figure S10.** HR-ESI-MS spectrum of **2**........................................................................................... S7

**Figure S11.** ^1^H-NMR spectrum (CDCl_3_, 500 MHz) of **2**................................................................. S8

**Figure S12.** ^13^C-NMR spectrum (CDCl_3_, 125 MHz) of **2**................................................................ S8

**Figure S13.** ^1^H-^1^H COSY spectrum of **2**. .................................................................................. ..... S9

**Figure S14.** NOESY spectrum of **2**....................................................................................... .......... S9

**Figure S15**. HMBC spectrum of **2** .................................................................................................S10

**Figure S16**. HSQC spectrum of **2**.................................................................................................. S10

**
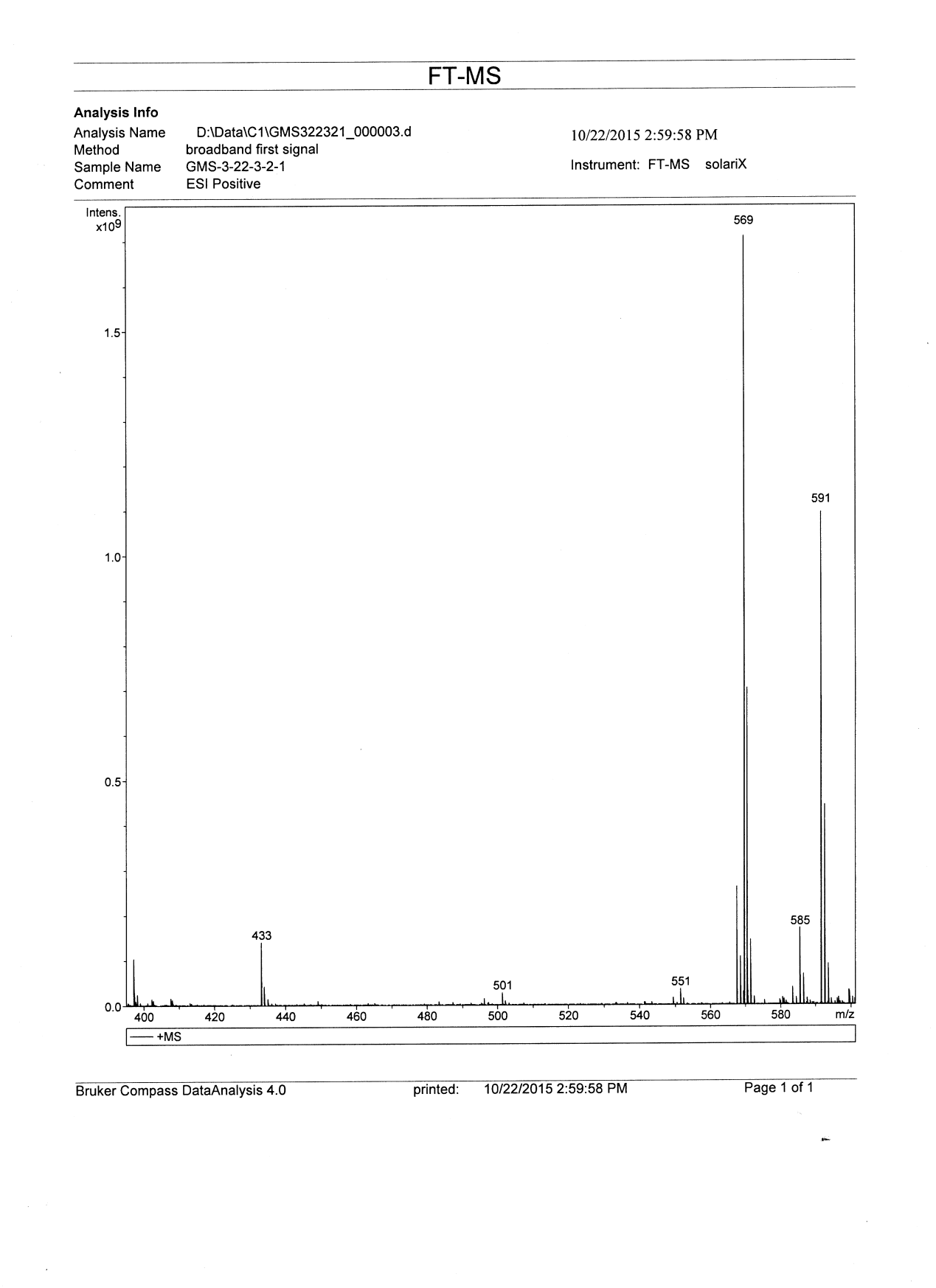
**

**Figure S1.** ESI-MS spectrum of **1**.


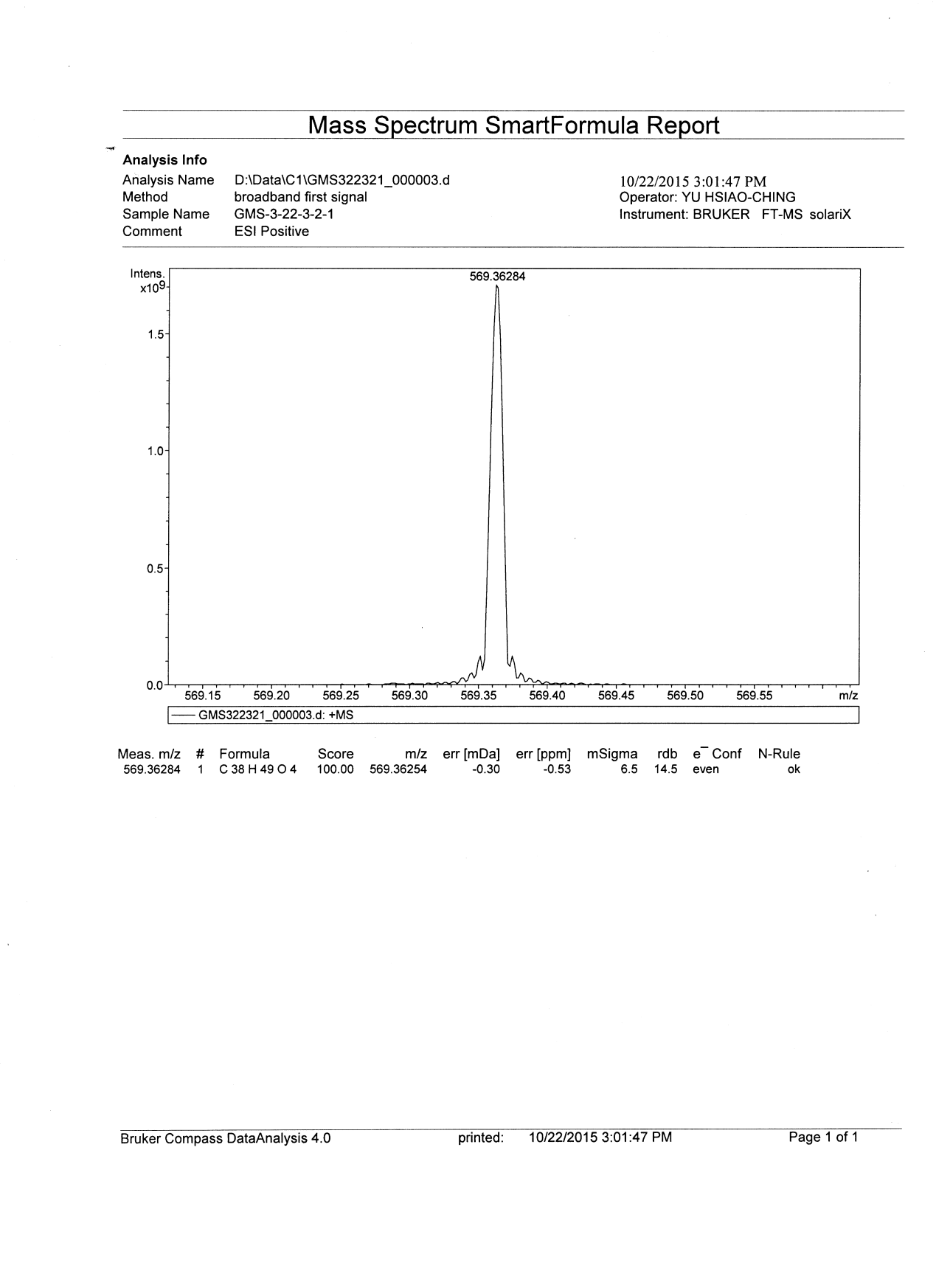


**Figure S2.** HR-ESI-MS spectrum of **1**.


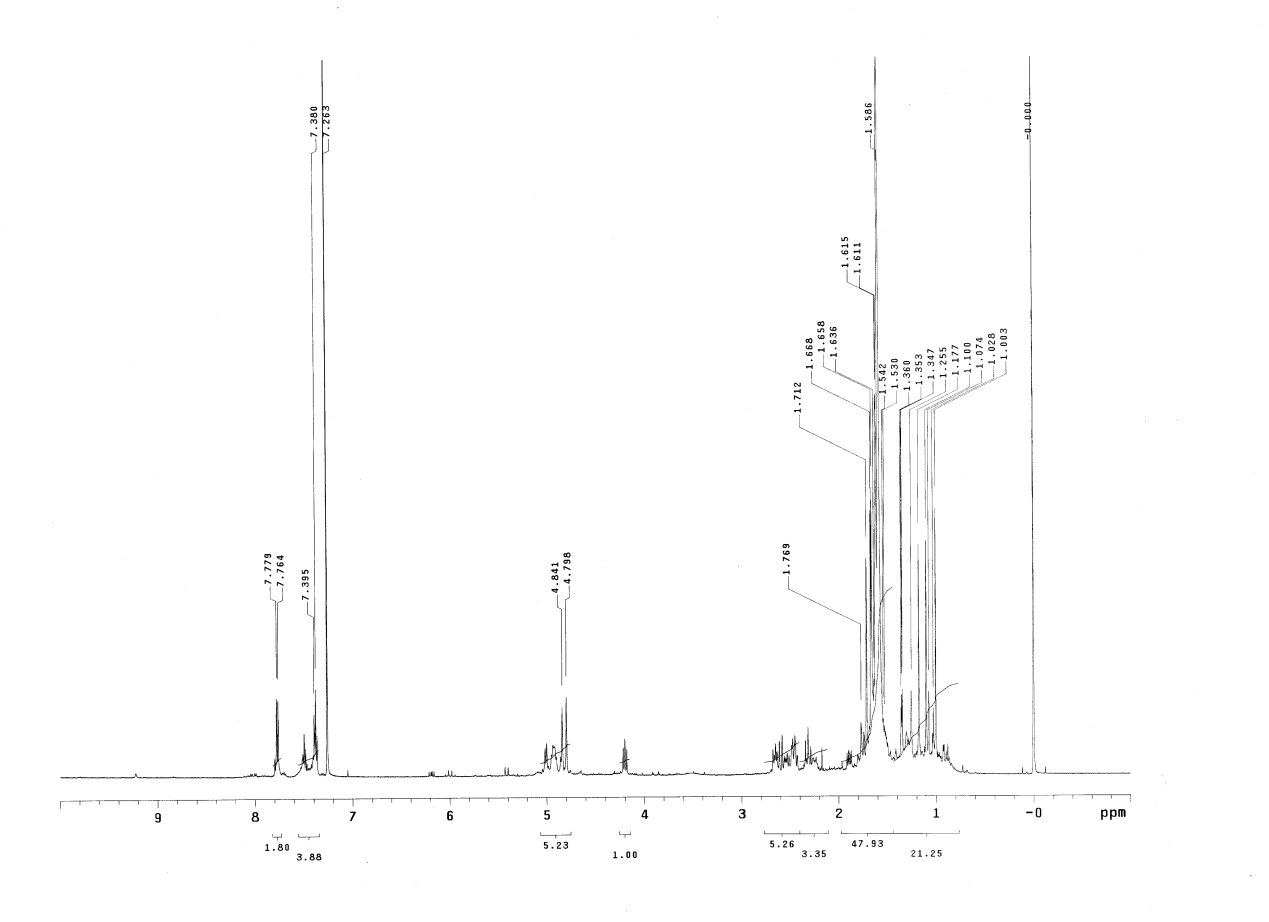


**Figure S3.** ^1^H-NMR spectrum of **1** (CDCl_3_ , 500 MHz).


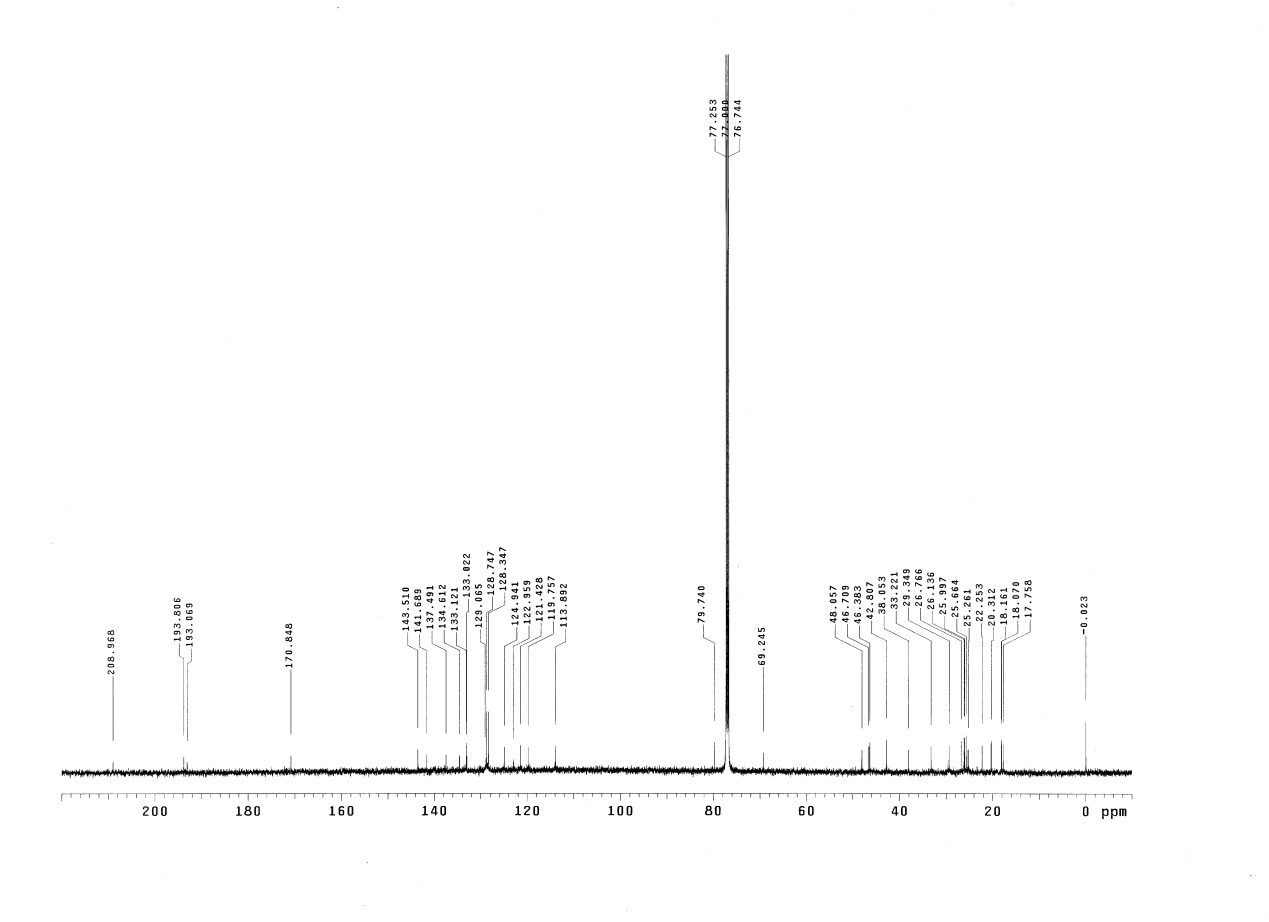


**Figure S4.** ^13^C-NMR spectrum of **1** (CDCl_3_, 125 MHz).

**
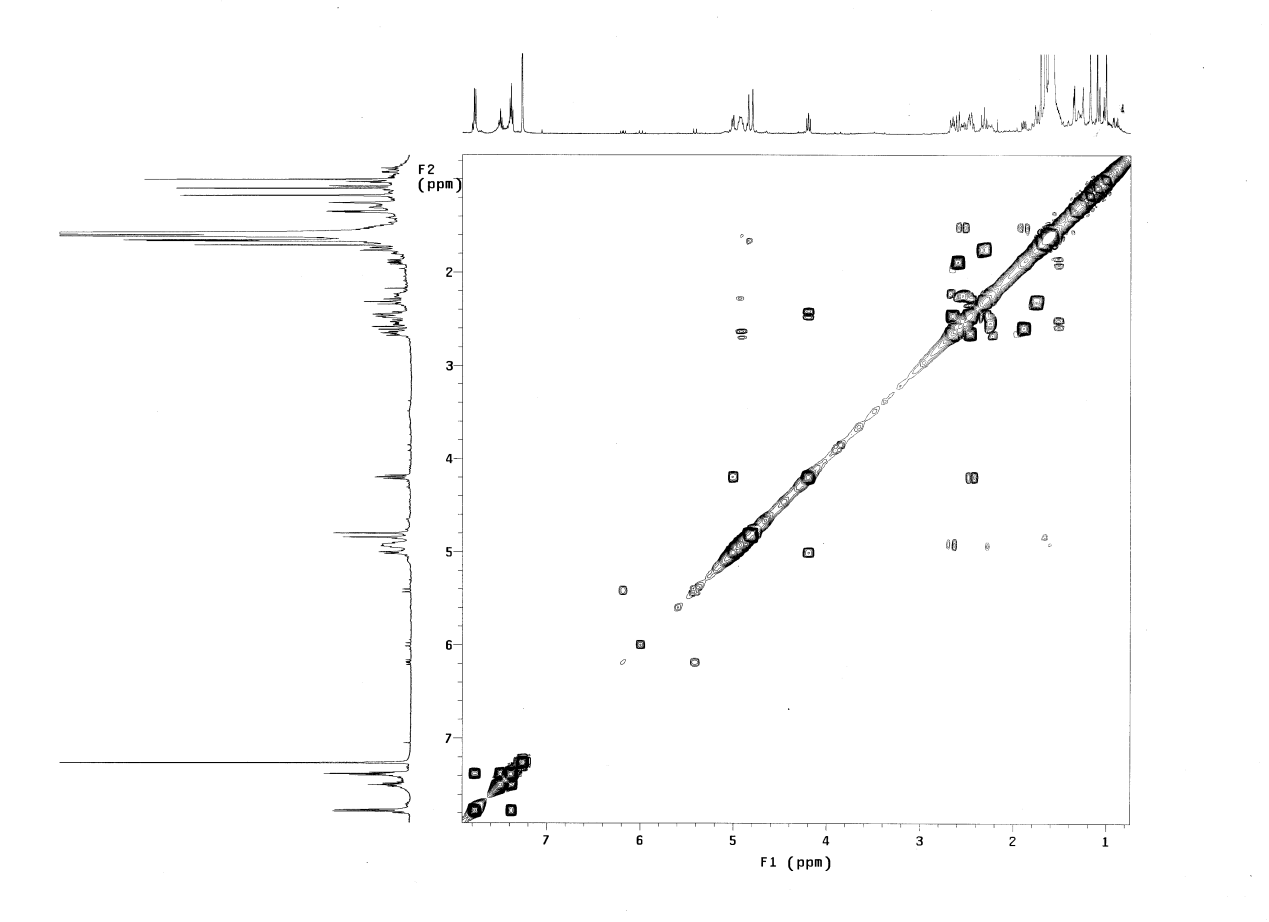
**

**Figure S5.** ^1^H–^1^H COSY spectrum of **1**.


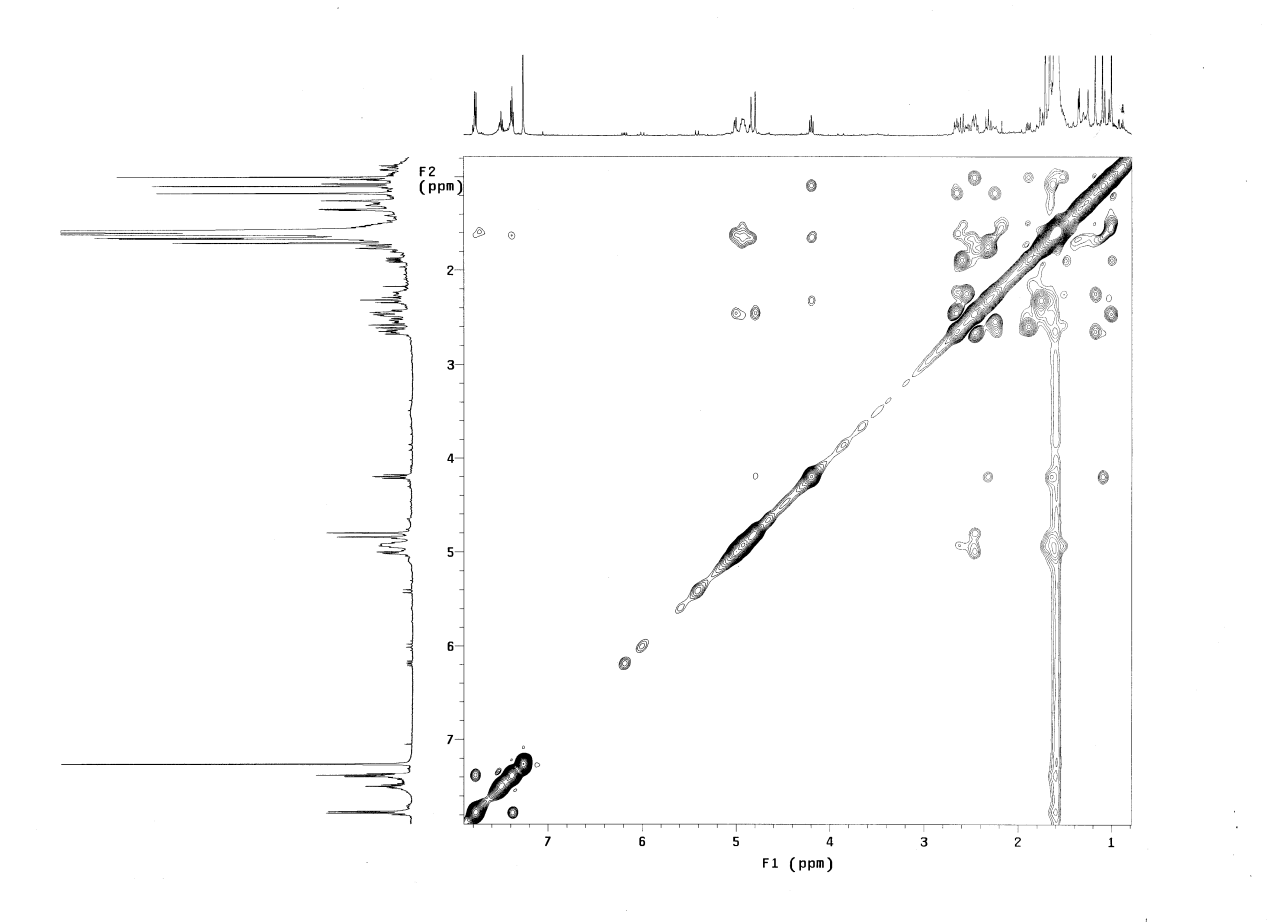


**Figure S6.** NOESY spectrum of **1.**

**
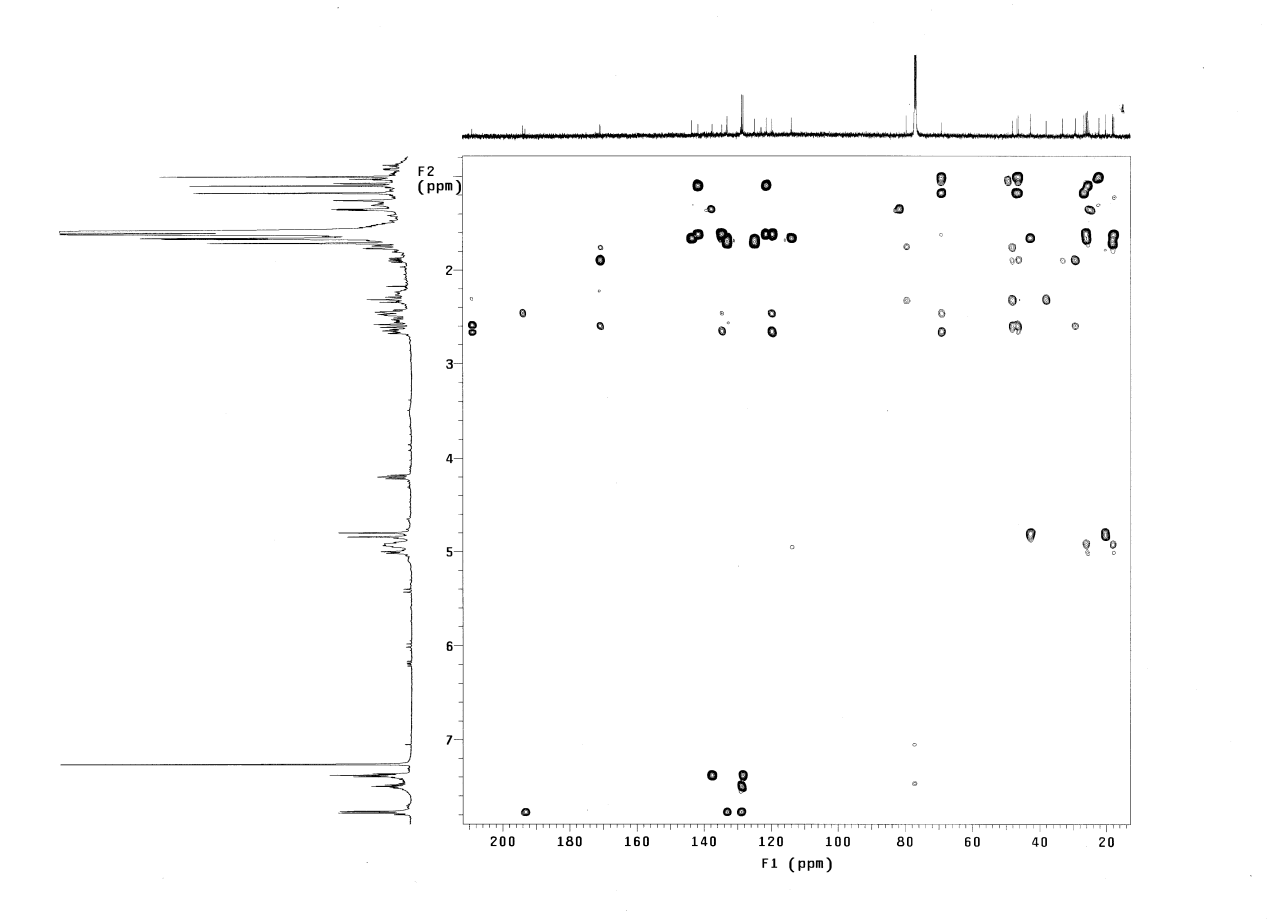
**

**Figure S7.** HMBC spectrum of **1**.


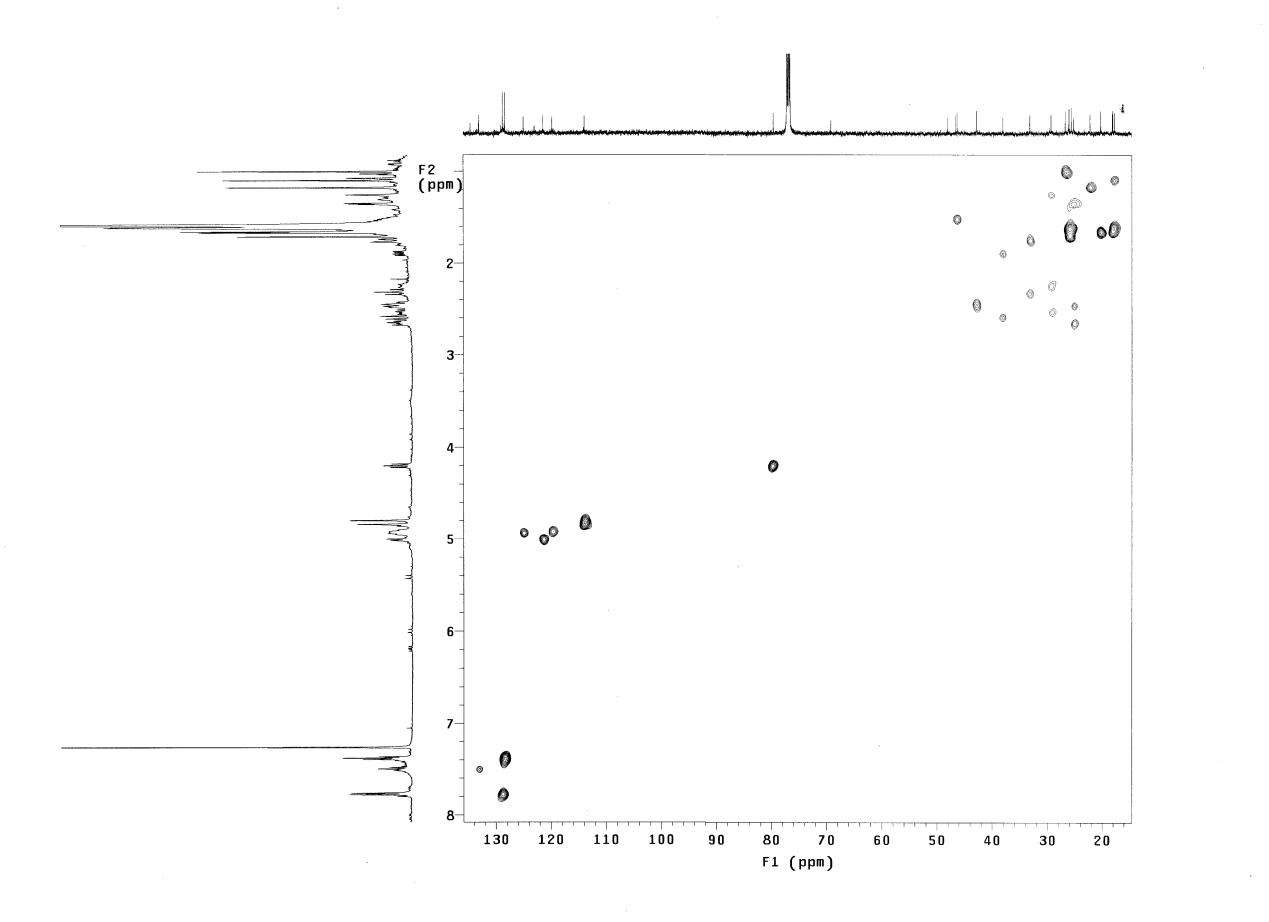


**Figure S8.** HSQC spectrum of **1**.


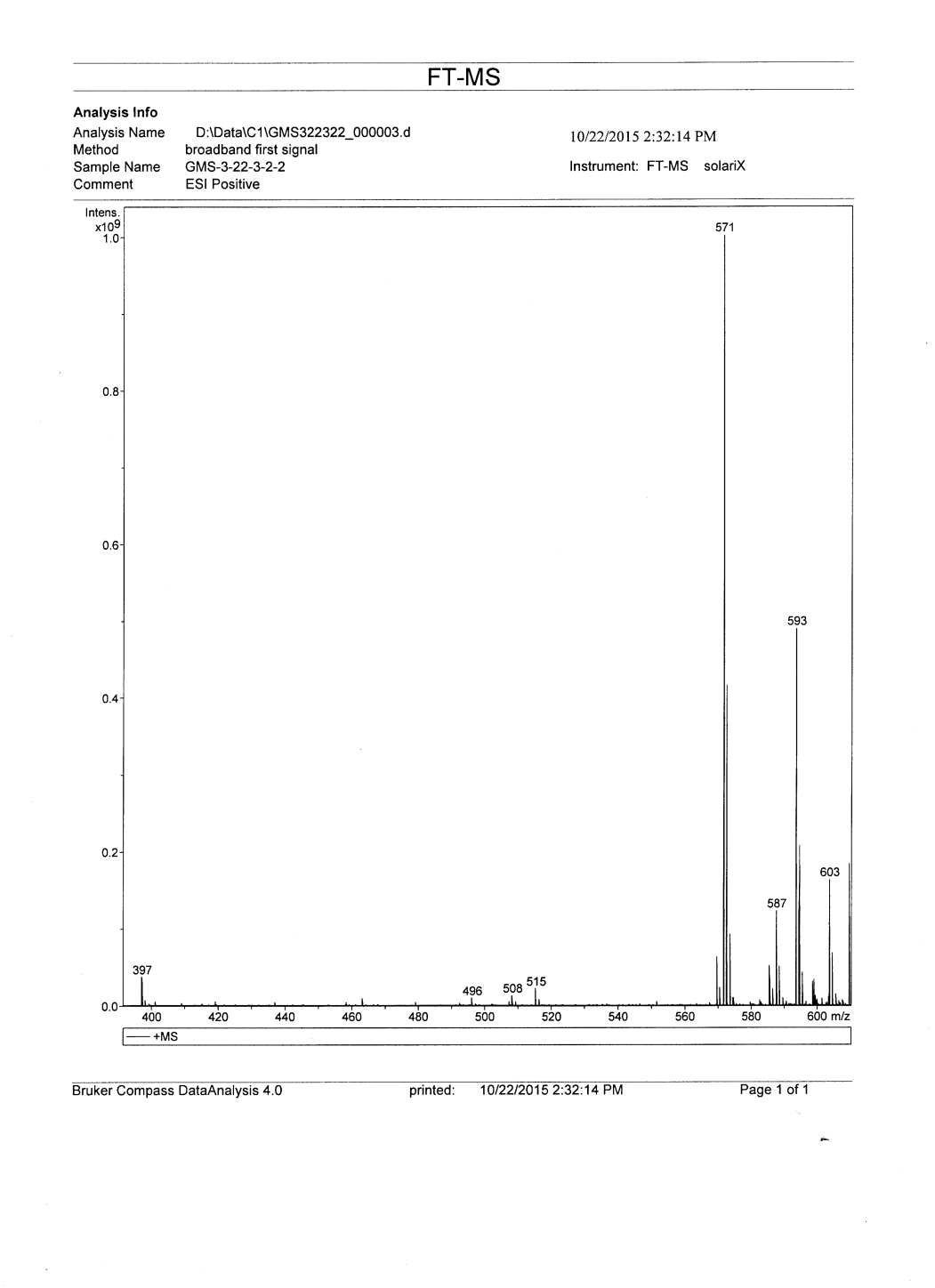


**Figure S9.** ESI-MS spectrum of **2**.

**
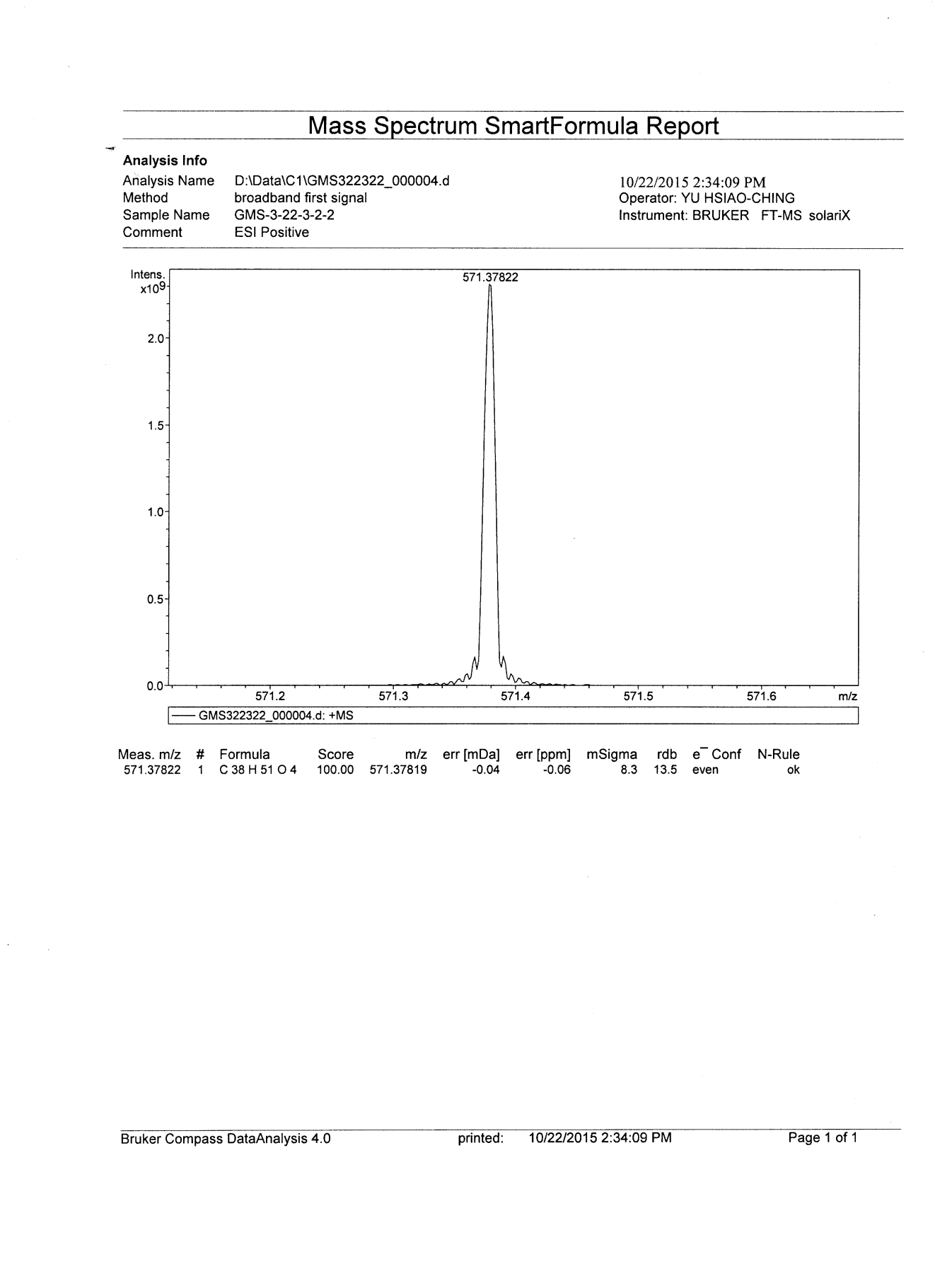
**

**Figure S10.** HR-ESI-MS spectrum of **2**.


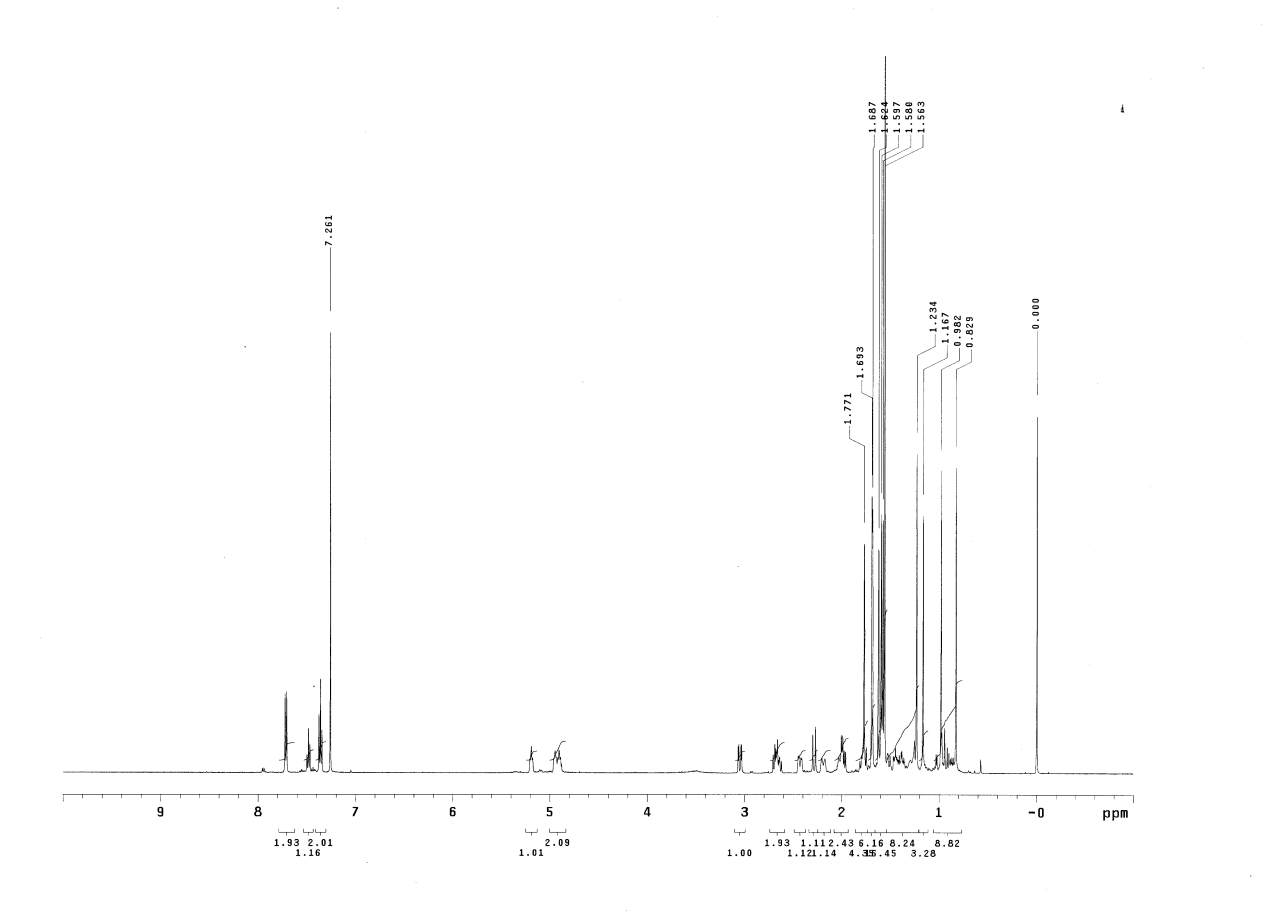


**Figure S11.** ^1^H-NMR spectrum of **2** (CDCl_3_, 500 MHz).


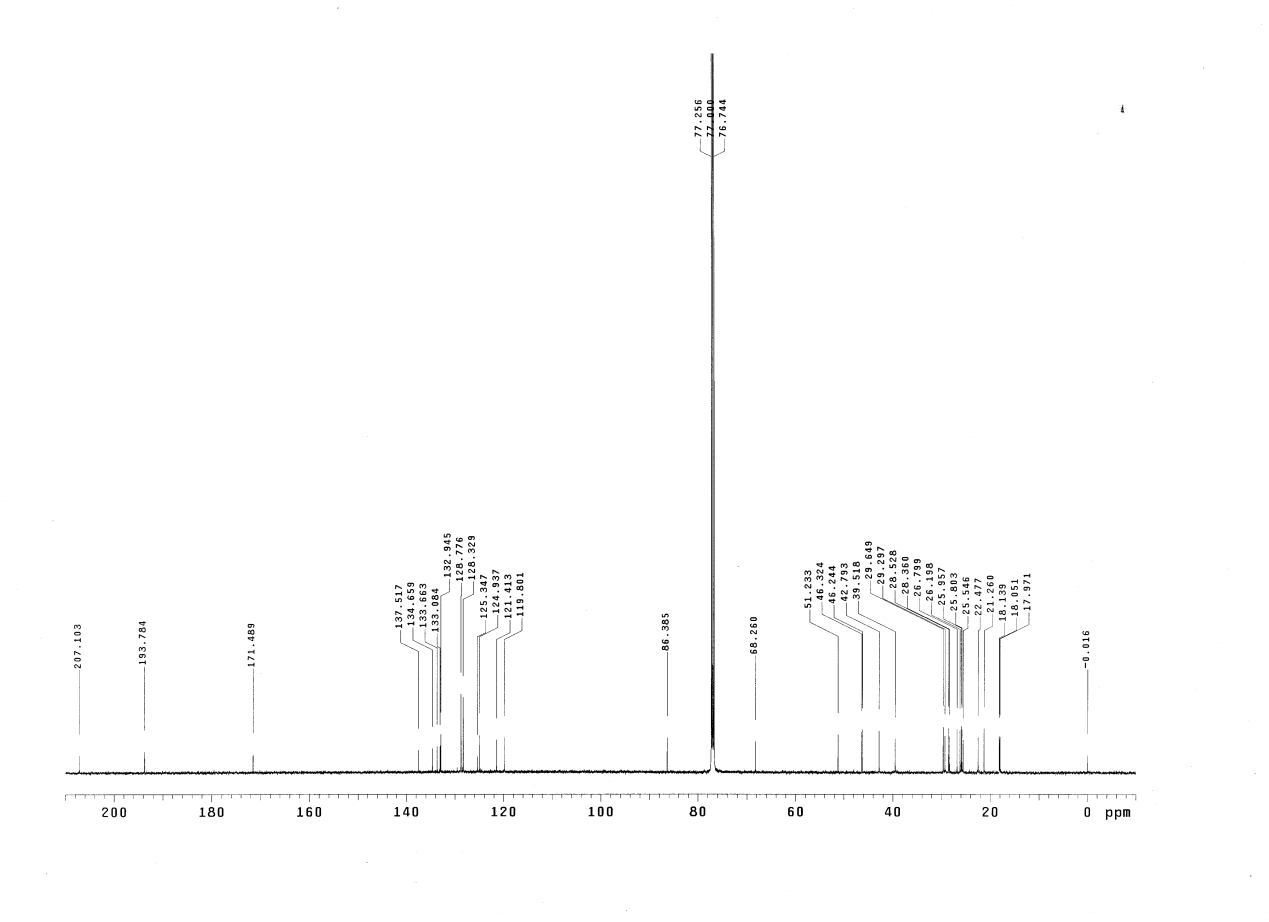


**Figure S12.** ^13^C-NMR spectrum of **2** (CDCl_3_, 125 MHz).


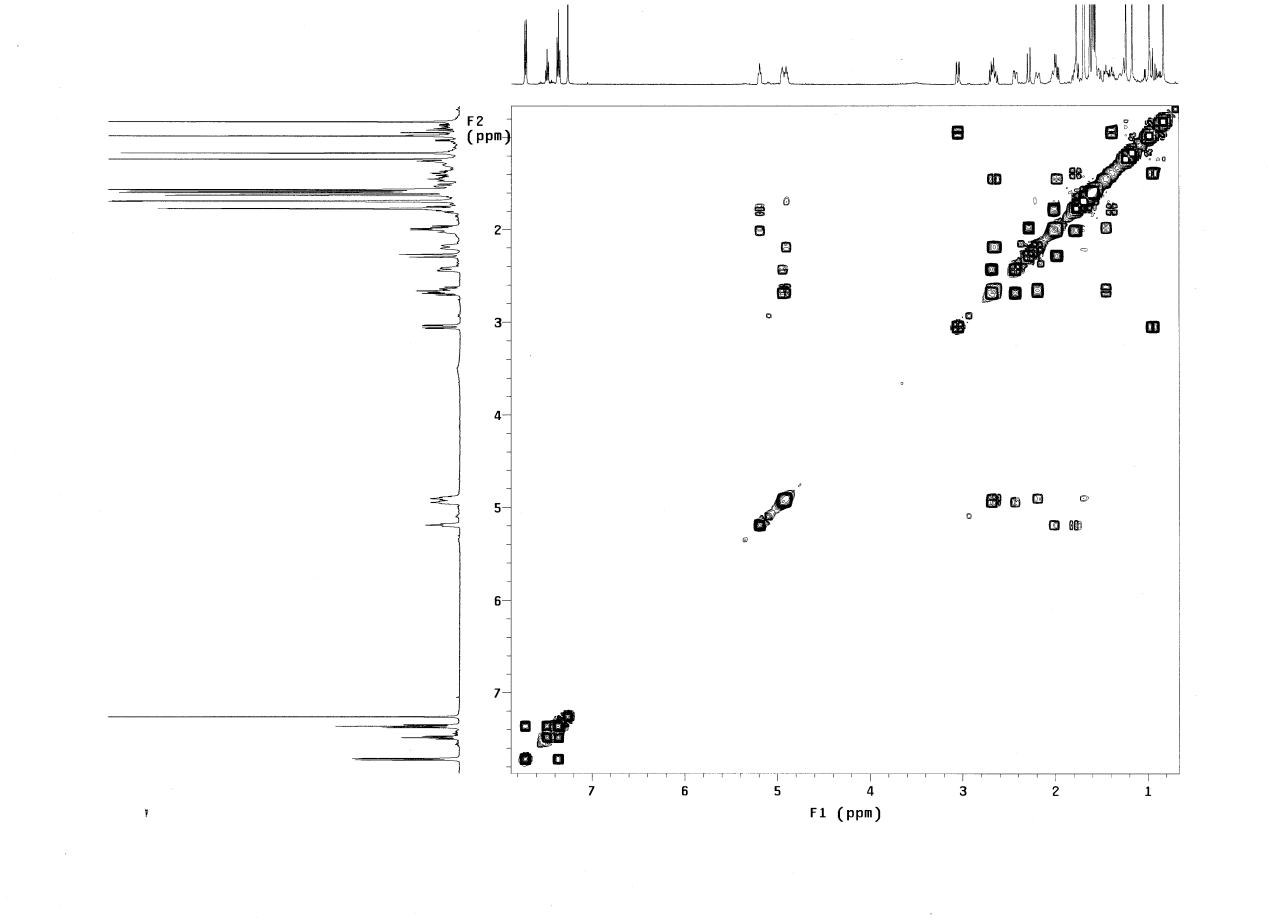


**Figure S13.** ^1^H–^1^H COSY spectrum of **2**.


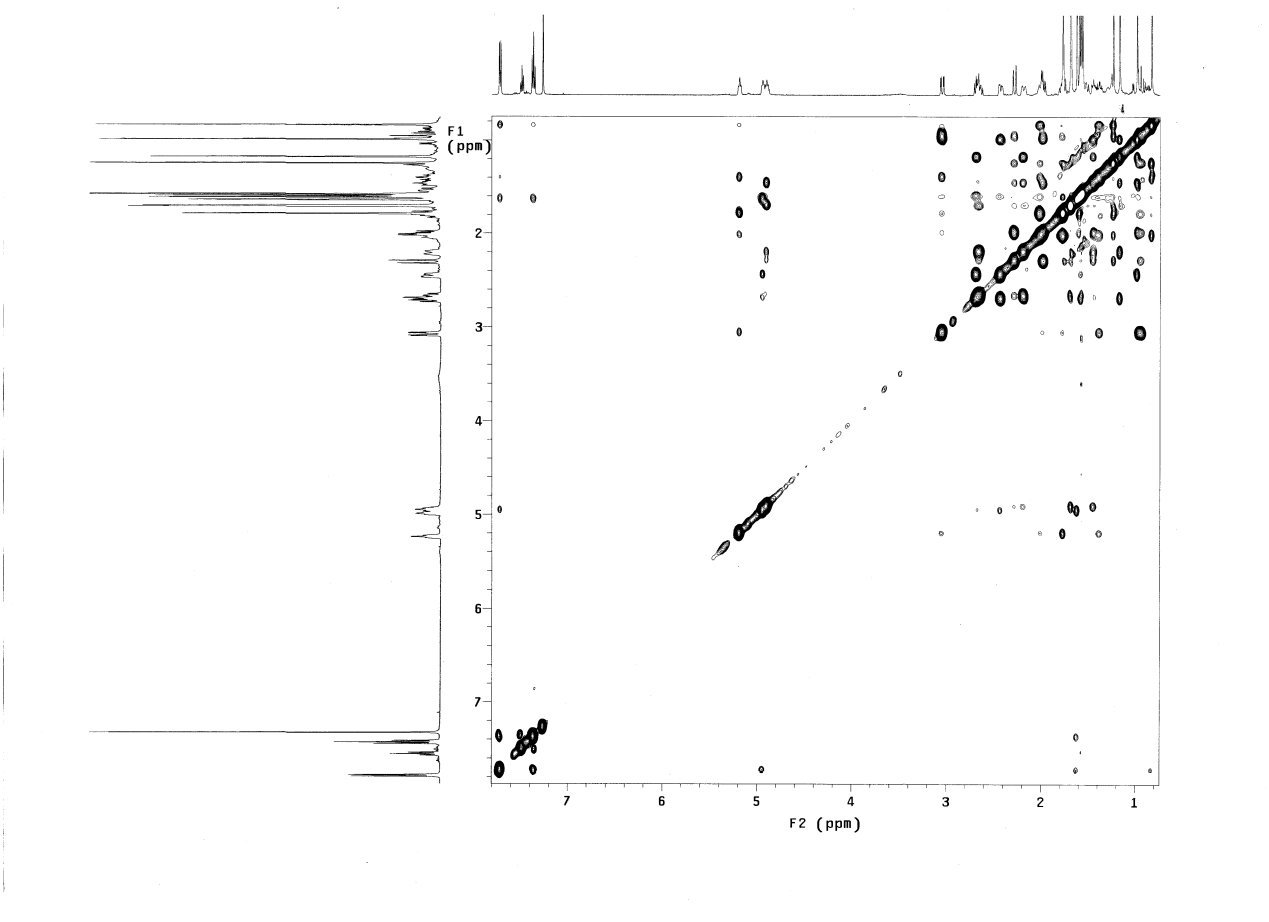


**Figure S14.** NOESY spectrum of **2**.


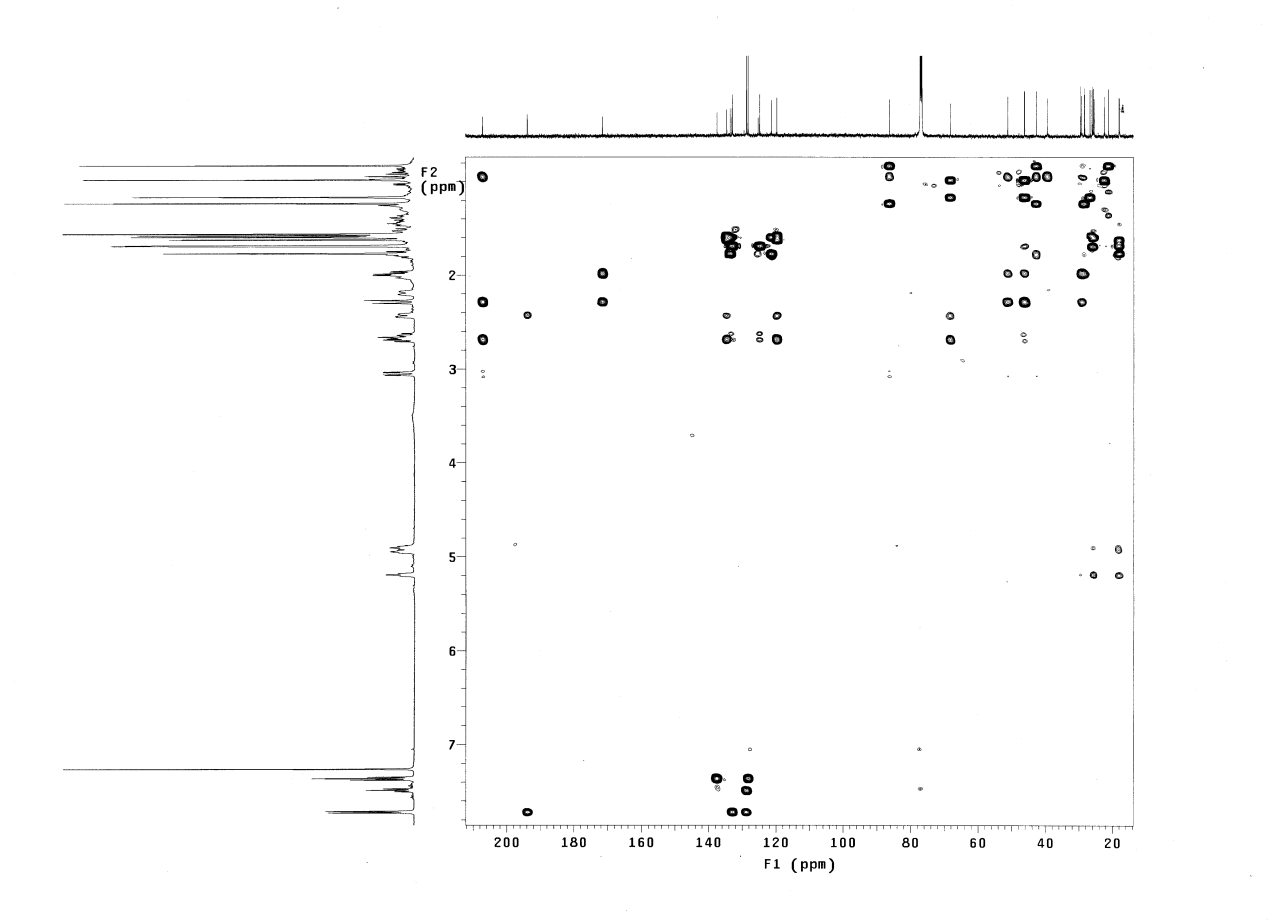


**Figure S15.** HMBC spectrum of **2**.


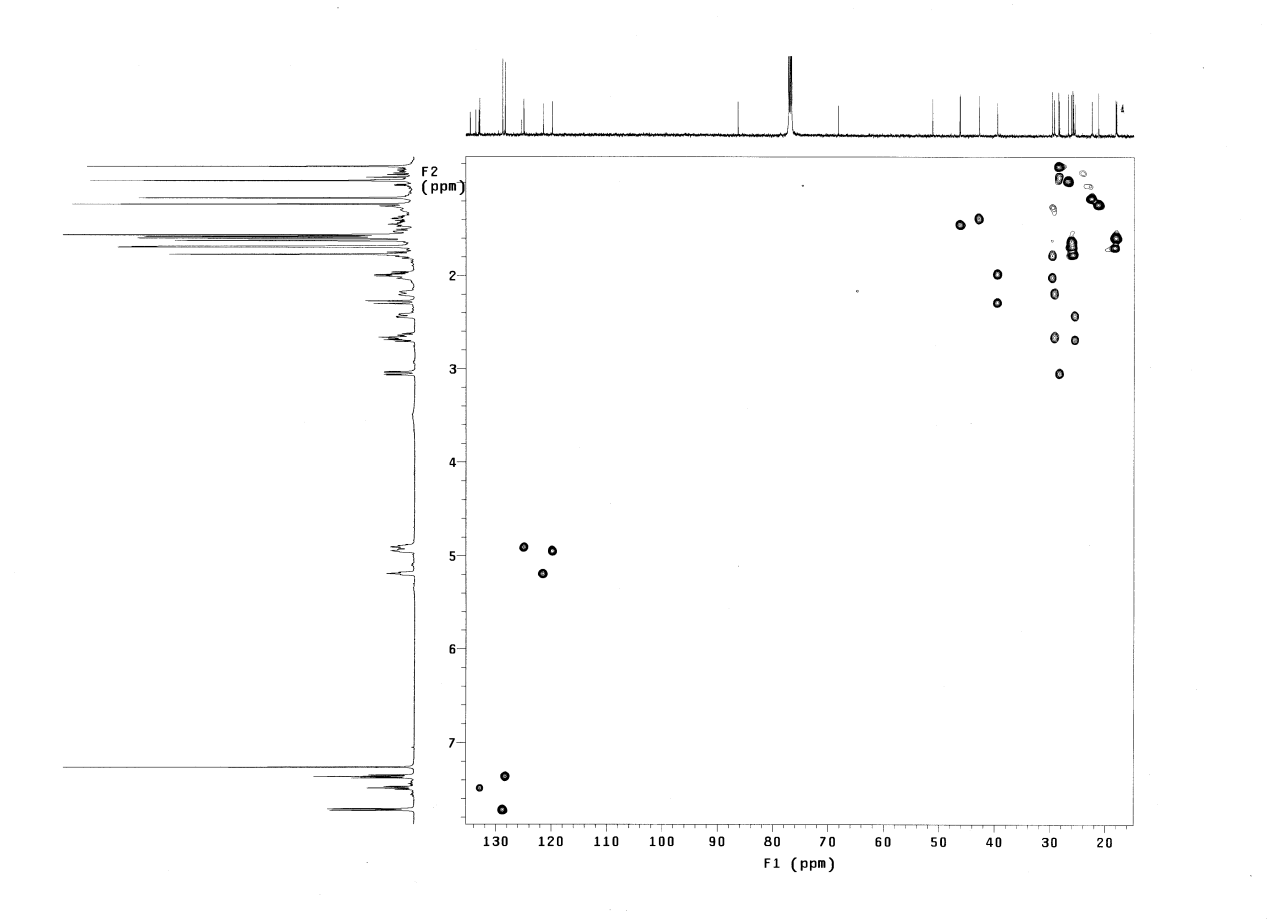


**Figure S16.** HSQC spectrum of **2**.
